# Supplementary material for: Constructs across a hierarchical, dimensional model of psychopathology show differential associations with social and general cognitive ability
Source: PLoS One. 2025 Jan 22;20(1):e0317377. doi: 10.1371/journal.pone.0317377 (PMC11753682; doi:10.1371/journal.pone.0317377)
Supplement: S1 File — This file contains supporting methodological information. (PDF) [file pone.0317377.s001.pdf]

Constructs Across a Hierarchical, Dimensional Model of Psychopathology Show Differential Associations with Social and General Cognitive Ability

Supplemental Information

Psychopathology Self-Report Measures

Personality Inventory for DSM-5 (PID-5). The PID-5 (Krueger et al., 2012) is a 220-item questionnaire that assesses 25 maladaptive personality trait facets that can be grouped into five categories (i.e., antagonism, detachment, disinhibition, negative affect, and psychoticism). Facet scales range from 4 to 14 items and are rated on a 4-point scale ranging from 1 ("very false or often false") to 4 ("very true or often true"). Participants in Samples 1 and 2 completed the full PID-5.

Schizotypal Personality Questionnaire (SPQ). The SPQ (Raine, 1991) assesses schizotypy—a multidimensional construct typically conceptualized as an intermediate phenotype associated with schizophrenia, schizotypal personality, and other psychotic disorders. The questionnaire consists of 74 yes/no items. Items are aggregated into nine subscales: ideas of reference, social anxiety, odd beliefs, unusual perceptual experiences, eccentric behavior, no close friends, odd speech, constricted affect, and suspiciousness. The SPQ was completed by Sample 1.

Externalizing Spectrum Inventory Brief Form (ESI-BF). The ESI-BF (Patrick et al., 2013) is a shortened version of the 415-item ESI, a measure of externalizing and its constituent subdimensions. This 160-item questionnaire assesses general disinhibition, substance abuse, callous aggression, and 23 lower-order facets of the externalizing spectrum. Participants rated each item on a 4-point scale, with higher scores corresponding to greater agreement with the

item. The current study used all lower-order facet scales from the General-Disinhibition and Callous Aggression domains. These scales were completed by Sample 2.

Social Cognition Measures

Tricky Triangles. In the triangles task (Abell et al., 2000; Barch et al., 2013; Castelli et al., 2002; White et al., 2011), participants are presented with a series of computerized animations of shapes interacting in a way that was random, physical, or social. In the random condition, the shapes did not interact with each other, but rather moved around purposelessly (e.g., bouncing or drifting). In the physical condition, the shapes moved in a goal-directed manner without invoking ToM or mentalizing (e.g., following one another). In the social condition, shapes enacted a social sequence, such as surprising, playing, or bullying. Participants were tasked with indicating whether each animation was random, physical, or social in nature, then scored for their accuracy. Accuracy was recorded as percent correct across conditions. Participants in Sample 1 completed a version of the task, described in detail by Demro and colleagues (2021), which was adapted from the original Human Connectome Project task-fMRI battery. Although participants completed this task during fMRI, only the behavioral data was considered in the present study.

Penn Emotion Recognition Task (ER-40). In the ER-40 (Gur, Ragland, Moberg, Bilker, et al., 2001; Gur, Ragland, Moberg, Turner, et al., 2001; Kohler et al., 2003), participants were shown a series of 40 face images, each of which showed a particular emotion. They were asked to select the appropriate emotion for each face, from the options of “Happy,” “Sad,” “Angry,” “Scared,” and “No Feeling.” Eight stimuli were presented for each of the emotion conditions. Participants’ accuracy was recorded and scored out of 40. Participants in Sample 1 completed this task.

Performance-based Prosody Identification Test (POSIT-PROID). We also administered the PROID test (Russ et al., 2008) from the POSIT Science Company's online test battery (see https://www.brainhq.com). This test of emotion recognition has participants listen to 21 auditory stimuli. For each trial, a recorded speaker reads a neutral sentence aloud in a way that is intended to convey one of six emotions (happiness, sadness, anger, fear, surprise, disgust). Participants were asked to identify each emotion and rate its intensity. A composite accuracy score based on how many stimuli each participant selected as the correct emotion category was used for the current analyses. Participants in Sample 1 completed this task.

Social Attribution Task-Multiple Choice (SAT-MC-II). For the SAT-MC-II (Johannesen et al., 2013, 2018), which is similar to the tricky triangles task, participants view an animation, which shows a triangle, oval, and rectangle acting out a social scene. The video is then presented in shorter segments, and participants answer a series of multiple-choice questions about each segment, which assess their ability to accurately attribute mental states. A total accuracy score was used for the current analyses. Participants in Sample 1 completed this task.

Mentalizing Vignettes. In this task, participants are asked to read a set of five short stories and answer a set of related true or false questions after each story (Stiller & Dunbar, 2007). Each story describes a social interaction involving multiple characters. After each story, participants answered five theory of mind (ToM) questions and five memory questions pertaining to the story, all in true-false format. Memory questions are designed to measure the participants' ability to retain the factual contents of the story, and the number of facts that the participant must retain varies from two to six in each question. Performance on memory questions within the task can be used as a covariate to ensure that any associations with variables of interest are due to participants' ToM ability rather than their memory for the details of the

story. ToM questions required that the participant reason, or infer, a character's perspective in the story. Questions vary across five levels of difficulty, with each successive level requiring the participant to track an additional character or level of perspective. For example, in second-level questions, participants tracked their own mental state and the mental state of one character (e.g., "John wanted to go home after work"). In fourth-level questions, participants tracked the mental state of three characters (e.g., "John thought that Penny knew what Sheila wanted to do"). To assess performance on the task, we adopted the procedure used by Allen et al. (2017) and computed simple sums of correct responses to memory questions and ToM questions for each participant. Participants in Sample 2 completed this task.

Supplemental References

Abell, F., Happé, F., & Frith, U. (2000). Do triangles play tricks? Attribution of mental states to animated shapes in normal and abnormal development. *Cognitive Development*, *15*(1), 1–16. https://doi.org/10.1016/S0885-2014(00)00014-9

Barch, D. M., Burgess, G. C., Harms, M. P., Petersen, S. E., Schlaggar, B. L., Corbetta, M., Glasser, M. F., Curtiss, S., Dixit, S., Feldt, C., Nolan, D., Bryant, E., Hartley, T., Footer, O., Bjork, J. M., Poldrack, R., Smith, S., Johansen-Berg, H., Snyder, A. Z., & Van Essen, D. C. (2013). Function in the human connectome: Task-fMRI and individual differences in behavior. *NeuroImage*, *80*, 169–189. https://doi.org/10.1016/j.neuroimage.2013.05.033

Castelli, F., Frith, C., Happé, F., & Frith, U. (2002). Autism, Asperger syndrome and brain mechanisms for the attribution of mental states to animated shapes. *Brain*, *125*(8), 1839–1849. https://doi.org/10.1093/brain/awf189

Gur, R. C. (2018). *"Precision Neuropsychology": Neuropsychological assessment in the "Precision Medicine" era*. International Neuropsychological Society. https://www.the-ins.org/files/meeting_ce/dc2018/CE_07_U3MrjGgEzS/CE7 Gur B_W.pdf

Gur, R. C., Ragland, J. D., Moberg, P. J., Bilker, W. B., Kohler, C., Siegel, S. J., & Gur, R. E. (2001). Computerized neurocognitive scanning: II. The profile of schizophrenia. *Neuropsychopharmacology*, *25*(5), 777–788. https://doi.org/10.1016/S0893-133X(01)00279-2

Gur, R. C., Ragland, J. D., Moberg, P. J., Turner, T. H., Bilker, W. B., Kohler, C., Siegel, S. J., & Gur, R. E. (2001). Computerized neurocognitive scanning: I. methodology and validation in healthy people. *Neuropsychopharmacology*, *25*(5), 766–776. https://doi.org/10.1016/S0893-133X(01)00278-0

Johannesen, J. K., Fiszdon, J. M., Weinstein, A., Ciosek, D., & Bell, M. D. (2018). The Social Attribution Task - Multiple Choice (SAT-MC): Psychometric comparison with social cognitive measures for schizophrenia research. *Psychiatry Research*, *262*, 154–161. https://doi.org/10.1016/j.psychres.2018.02.011

Johannesen, J. K., Lurie, J. B., Fiszdon, J. M., & Bell, M. D. (2013). The Social Attribution Task-Multiple Choice (SAT-MC): A psychometric and equivalence study of an alternate form. *International Scholarly Research Notices*, *2013*, e830825. https://doi.org/10.1155/2013/830825

Kohler, C. G., Turner, T. H., Bilker, W. B., Brensinger, C. M., Siegel, S. J., Kanes, S. J., Gur, R. E., & Gur, R. C. (2003). Facial emotion recognition in schizophrenia: Intensity effects and error pattern. *American Journal of Psychiatry*, *160*(10), 1768–1774. https://doi.org/10.1176/appi.ajp.160.10.1768

Krueger, R. F., Derringer, J., Markon, K. E., Watson, D., & Skodol, A. E. (2012). Initial construction of a maladaptive personality trait model and inventory for DSM-5. *Psychological Medicine*, *42*(9), 1879–1890. https://doi.org/10.1017/S0033291711002674

Patrick, C. J., Kramer, M. D., Krueger, R. F., & Markon, K. E. (2013). Optimizing efficiency of psychopathology assessment through quantitative modeling: Development of a brief form of the Externalizing Spectrum Inventory. *Psychological Assessment*, *25*, 1332–1348. https://doi.org/10.1037/a0034864

Raine, A. (1991). The SPQ: A Scale for the assessment of schizotypal personality based on DSM-III-R criteria. *Schizophrenia Bulletin*, *17*(4), 555–564. https://doi.org/10.1093/schbul/17.4.555

Russ, J. B., Gur, R. C., & Bilker, W. B. (2008). Validation of affective and neutral sentence content for prosodic testing. *Behavior Research Methods*, *40*(4), 935–939. https://doi.org/10.3758/BRM.40.4.935

Stiller, J., & Dunbar, R. I. M. (2007). Perspective-taking and memory capacity predict social network size. *Social Networks*, *29*(1), 93–104. https://doi.org/10.1016/j.socnet.2006.04.001

White, S. J., Coniston, D., Rogers, R., & Frith, U. (2011). Developing the Frith-Happé animations: A quick and objective test of theory of mind for adults with autism. *Autism Research*, *4*(2), 149–154. https://doi.org/10.1002/aur.174
