# Supplementary material for: Constructs across a hierarchical, dimensional model of psychopathology show differential associations with social and general cognitive ability
Source: PLoS One. 2025 Jan 22;20(1):e0317377. doi: 10.1371/journal.pone.0317377 (PMC11753682; doi:10.1371/journal.pone.0317377)
Supplement: Attachment — Submitted filename: PlosOne_ReviewResponses_Final.docx [file pone.0317377.s002.docx]

**We thank the editor and reviewers for their thoughtful responses to our paper and for their overall positive reception of our work. We have revised our paper as follows, responding to each of the relevant critiques. Revisions are also noted in the main text, using red. We appreciate *PLOS One’s* continued consideration of our work and look forward to the opportunity to publish our research in the journal.**

Reviewer #1: In this well-performed and interesting study, the authors examine dimensions of psychopathology and associations with social and general cognitive ability.
The study is relevant in exploring the dimensional approach to psychopathology using the HiTOP model, and the analyses are golden standard. There are some minor considerations, mainly pertaining to data quality and presentation of results:

1. The authors mention themselves in the limitation section the important issue with the quality of data, such as the use of only one social cognitive measure in Sample 1 (Mentalizing Vignettes), which comprises other subdomains than those used in Sample 2; the use of self-report questionaries to measure dimensions of psychopathology; and recruiting related issues / sample differences (sign. different age e.g.). The study appear with a pragmatic use of existing data pooled from other original studies, and must be very clear in this explorative basis. Although hypothesis are introduced, the data collection were not designed for the current study. Following, any results and conclusions must be clearly stated cautious, not generalizable and only hypothesis generating for further examination. Furthermore, the difference in social cognitive measures may not only contribute to inconsistent associations as stated - but also consistent associations may be a random effect, as we cannot be sure the same concepts are measured.

**R1. We have further highlighted the partially exploratory and tentative nature of our current findings. We now spend greater time discussing these issues raised by the reviewer, in our discussion section, as quoted below.**

“Given these limitations, our findings should be interpreted as preliminary. Future research should be conducted to confirm the present results in a large sample with data designed specifically to assess relations among psychopathology dimensions, general cognitive ability, and social cognitive ability (across multiple domains in the same participants). This would allow us to determine whether the present findings are robust and generalizable.”

2. I do miss an attempt to clarify in post hoc testing the more specific dynamic effect of GCA in the relation between p-factor and social cognition. Is it potentially mediating or moderating the effect?

**R2. In our original findings, we did test for a statistical mediation effect and in fact found evidence for significant indirect effects of p-factor on social cognition through general cognitive ability (whereas direct effects of Detachment on social cognition were significant). Technically, we did not find statistical mediation (as typically discussed), as the total effect of p-factor on social cognition was not significant. We also wanted to avoid using strong language around “mediation”, given that many individuals interpret this language causally but our current study design is not causally informative. Refer to the “direct” vs. “indirect” effects in Tables 7 and 8 to see results relevant to statistical mediation.**

**We did not originally test for interactions, given the large number of variables in our dataset and limitations of MPlus for simultaneously estimating exploratory measurement factors and interaction effects. We have added post-hoc, exploratory analyses to test for interactions, as noted below.**

“[F]ollow-up analyses were conducted for each sample. First, we conducted post-hoc, exploratory moderation analyses to examine whether any psychopathology variables significantly interacted with general cognitive ability when statistically predicting social cognition. To do so, we extracted estimated factor scores for psychopathology dimensions and general cognitive ability (as well as for Social Cognition in the P-HCP dataset) using the regression method in Mplus. Interaction terms were then computed for each psychopathology dimension by multiplying corresponding factor-score estimates with the factor-score estimates for general cognitive ability. Then, we conducted regression analyses statistically predicting social cognition using general cognitive ability, each psychopathology factor score, and each of the general cognitive ability-by-psychopathology interaction terms.”

“In Sample 1, there were no significant interaction effects of psychopathology and general cognitive ability, in statistically predicting social cognition. In Sample 2, there was a significant negative interaction between general cognitive ability and dependability in statistically predicting social cognition (*β* = -.108, *p* = .019); given that both general cognitive ability and dependability positively predicted social cognition, a negative interaction indicates an attenuation of these positive effects given increases in the other respective variable.”

3. The should be separate analyses for sex, as this may be important for both psychopathology and social cognition.

**R3. We have added additional analyses including sex and psychopathology-by-sex interactions, which are now incorporated into our methods, results, and discussion, as quoted below.**

“[F]ollow-up analyses were conducted for each sample… [W]e conducted post-hoc regression analyses using… extracted factor scores to confirm whether key findings remained significant when controlling for sex and to examine whether there were any significant psychopathology-by-sex interactions in statistically predicting social cognition. In these analyses, sex was included as a dummy-coded predictor variable (females = 0, males = 1). Interaction terms were then computed for each psychopathology dimension by multiplying corresponding factor-score estimates by the sex dummy variable. Then, we conducted regression analyses statistically predicting social cognition using general cognitive ability, sex, each psychopathology factor score, and each of the sex-by-psychopathology interaction terms. Simple-slopes tests were then done to further examine any significant psychopathology-by-sex interactions.”

“In both samples, all effects identified as significant in our primary ESEM analyses remained significant when controlling for sex (and directions of effects remained consistent). When accounting for general cognitive ability and psychopathology, Females showed better social cognition than males in both Samples 1 (β = -.066, p = .008) and 2 (β = -.104, p = .034).

In Sample 1, there was a significant, negative interaction between sex and Emotionality (β = -.053, p = .035). Given that females performed better on social cognition and higher Emotionality was marginally associated with worse social cognition, this negative interaction appears to indicate that the association between Emotionality and worse social cognition may be particularly prominent in males. Indeed, follow-up simple-slope tests suggest that males show a fairly strong association between Emotionality and worse social cognition (β = -.284, p < .001), females show a weaker negative association (β = -.091, p = .027).

In Sample 2, there was a significant, positive interaction between sex and Detachment (β = .128, p = .008). Given that females performed better on social cognition and higher Detachment was associated with worse social cognition, this positive interaction appears to indicate that the association between Detachment and worse social cognition may be particularly prominent in females. Indeed, follow-up simple-slope tests suggest that females showed a significant association between Detachment and worse social cognition (β = -.247, p < .001), whereas the association was not significant in males (β = -.026, p = .694).”

“In addition to these primary contributions, our findings also echo previous work that shows superior social cognitive ability in females [30,53,63] and offer preliminary findings to inform future research on how sex and psychopathology may interact to explain variance in social functioning.”

A few minor comments:
4. Rhetorical questions in an introduction is a really bad style.

**R4. We have revised our first paragraph to now avoid use of rhetorical questions.**

**“**Humans are inherently social, yet there is significant variation in our ability and motivation to form and maintain relationships. Some individuals navigate complex social situations—like networking, dating, and interviewing—with ease, while others find even simple interactions—such as visiting a coffee shop or making a phone call—a challenge. Investigating individual differences in the mechanisms that facilitate social interaction provides insight into this variation. For instance, social cognitive processes—including emotion recognition and mentalizing—enable individuals to recognize and interpret the mental and emotional states of others. These processes are crucial for adaptive functioning; however, individuals with mental illness or at higher risk of psychopathology often experience difficulties in one or more of these social cognitive domains.”

5. ASD is not a psychiatric disorder, but a neurodevelopmental disorder. The distinction is important for ASD subjects.

**R5. We have revised our language accordingly to reflect the standard terminology of DSM-5.**

“Research has consistently reported social cognitive deficits across a broad range of mental disorders—including schizophrenia, personality disorders, anxiety, and depression—and autism spectrum disorder [1–5].”

Reviewer #2: PONE-D-24-24635
Constructs Across a Hierarchical, Dimensional Model of Psychopathology Show Differential Associations with Social and General Cognitive Ability

EVALUATION

This is really interesting research addressing the relationships between social cognition and psychopathology studying two samples (Psychosis Human Connectome Project, P-HCP, and Twin cities –community sample).

Importantly, the role of cognitive ability (aka, intelligence) is also considered.

Furthermore, a latent variable approach is applied here.

One of the key findings is that the relationship between social cognition and the p factor can be accounted for by cognitive ability, while detachment is related with social cognition even after controlling for cognitive ability.

Tables 7 and 8 depict important findings that deserve attention.

Comparison among values obtained from both samples are highly informative. Thus, for instance, the -.30 value between general cognitive ability and the p factor in the P-HCP samples is in high contrast with the null value for this relationship in the Twin cities sample.

The general picture provides important information.

The Ms. is well written and goes to the heart of the main research questions.

Therefore, I recommend publication.

**R6. We are excited to hear Reviewer 2’s positive reception of our work! We hope that our revisions in response to Reviewer 1 have made the paper even stronger.**

**In addition to these revisions in response to the reviewers, we have added the following statement regarding funding, as requested:**

“The funders had no role in data collection or analysis, decision to publish, or preparation of the manuscript. In reference to Sample 1, the NIMH did play some role in setting forth the study aims and study design—though not for these particular research questions and analyses—given that data collection was funded through a U01 cooperative agreement. For Sample 2 and for our particular set of analyses, the funders had no role in study design, data collection, analysis, decision to publish, or preparation of the manuscript.”
